# Addition of plant dietary fibre to a raw red meat high protein, high fat diet, alters the faecal bacteriome and organic acid profiles of the domestic cat (*Felis catus*)

**DOI:** 10.1371/journal.pone.0216072

**Published:** 2019-05-01

**Authors:** Christina F. Butowski, David G. Thomas, Wayne Young, Nick J. Cave, Catherine M. McKenzie, Douglas I. Rosendale, Emma N. Bermingham

**Affiliations:** 1 Food & Bio-based Products, AgResearch Grasslands, Palmerston North, New Zealand; 2 Centre for Feline Nutrition, Massey University, Palmerston North, New Zealand; 3 Riddet Institute, Massey University, Palmerston North, New Zealand; 4 High-Value Nutrition National Science Challenge, Auckland, New Zealand; 5 Knowledge & Analytics, AgResearch Grasslands, Palmerston North, New Zealand; 6 The New Zealand Institute for Plant & Food Research Ltd., Palmerston North, New Zealand; University of Illinois, UNITED STATES

## Abstract

Commercial diets high in animal protein and fat are increasingly being developed for pets, however little is understood about the impacts of feeding such diets to domestic cats. The carbohydrate content of these diets is typically low, and dietary fibre is often not included. Dietary fibre is believed to be important in the feline gastrointestinal tract, promoting stool formation and providing a substrate for the hindgut microbiome. Therefore, we aimed to determine the effects of adding plant-based dietary fibre to a high animal protein and fat diet. Twelve domestic short hair cats were fed three complete and balanced diets in a cross-over design for blocks of 21 days: raw meat (Raw), raw meat plus fibre (2%, ‘as is’ inclusion of inulin and cellulose; Raw+Fibre) and a commercially available Kibble diet. A commercially available canned diet was fed for 21 days as a washout phase. Apparent macronutrient digestibility, faecal output, score, pH, organic acid concentrations and bacteriome profiles were determined. Diet significantly affected all faecal parameters measured. The addition of dietary fibre to the raw meat diet was found to reduce apparent macronutrient digestibility, increase faecal output, pH and score. Thirty one bacterial taxa were significantly affected by diet. *Prevotella* was found to dominate in the Kibble diet, *Clostridium* and *Fusobacterium* in the Raw diet, and *Prevotella* and a group of unclassified *Peptostreptococcaceae* in the Raw+Fibre diet. Our results show that diets of different macronutrient proportions can strongly influence the faecal microbiome composition and metabolism, as shown by altered organic acid concentrations and faecal pH, in the domestic cat. The addition of 2% of each fibre to the Raw diet shifted faecal parameters closer to those produced by feeding a Kibble diet. These results provide a basis for further research assessing raw red meat diets to domestic cats.

## Introduction

Domestic cats are obligate carnivores and require relatively large amounts of protein and fat in their diet. Commercial pet foods, such as kibble diets, usually contain large quantities of carbohydrate (CHO), typically 46–74% on a DM basis [1], of which a small proportion (>4% DM) of this is dietary fibre. The feeding of diets high in animal protein and fats, with typically little or no dietary fibre (plant- or animal-derived), continues to increase in popularity [2]. However, very little research has been conducted investigating the impacts of feeding such diets to domestic cats.

Dietary fibre is defined as a substrate that is not digested in the small intestine but is instead partially or completely fermented in the large intestine [3, 4]. There are various types of dietary fibre which can most easily be categorised into fermentative and non-fermentative fibres, based on their physical and chemical properties. The National Research Council (NRC) states safe upper limits for some fibres included in pet food, but there is no minimum fibre requirement [1]. Similarly, American Association of Feed Control Officials (AAFCO) and Fédération européenne de l’industrie des aliments pour animaux familiers (FEDIAF) guidelines, do not prescribe a rate of fibre inclusion [5, 6]. The inclusion of dietary fibre in a human diet is thought to have beneficial effects, mainly due to its effect on the gastrointestinal microbiome and fermentation end products produced [7, 8].

Although cats are obligate carnivores, it has been noted that wild felids consume the hair, bone and skin of their prey that may act as a source of dietary fibre [9, 10]. When incorporated into extruded diets, insoluble, non-fermenting fibres such as cellulose, have been shown to alter faecal composition [11] and decrease apparent macronutrient digestibility [12, 13]. Fermentable fibres such as fructooligosaccharide and inulin, have also been shown to increase the production of fermentative end products and modify the colonic microbiota in cats fed extruded diets [11, 14]. Previous studies have assessed the inclusion of plant dietary fibre in raw red meat diets. Beloshapka et al [15] investigated inulin and yeast cell wall extract in dogs and have noted changes to the faecal microbiome, including increases in *Bifidobacterium* and *Lactobacillus*. Kerr et al [16] investigated cellulose and beet pulp inclusion in raw beef-based diets in captive exotic felids and observed increased faecal output and greater apparent total tract crude protein and fat digestibility in those fed cellulose. Therefore, the inclusion of a plant dietary fibre source to a raw diet fed to domestic cats may also exert similar effects.

The microbiome composition and activity is associated with many factors, including health status, age and diet [17–20] and plays a vital role in the host, in part through the fermentation of undigested dietary components, such as plant dietary fibre [21]. This produces fermentative end products, such as organic acids, which include short chain fatty acids (SCFA; acetate, butyrate and propionate), lactate, succinate, and branched chain fatty acids (BCFA; isobutyrate and isovalerate). SCFA are predominantly produced during carbohydrate fermentation, whereas the BCFA typically arise from protein fermentation [22]. Diet, the microbiome and consequent organic acid production/utilisation, modulates the colonic physiology and environment. For example, SCFAs effect colonic pH and alter the microbial community through changes to substrate provision [23]. In addition, they influence intestinal immunity [24] and motility [25], regulate sympathetic neuronal activity via G protein coupled receptors [26], and provide fuel for colonocytes [27]. The effect of the BCFA within the colon has not been thoroughly explored, but recent research has indicated a role in glucose and lipid metabolism [28]. Therefore, understanding the changes in the microbiome, (specifically the bacteriome (the gastrointestinal bacterial community)), and subsequent changes to organic acid production when cats are fed these diets, is of great interest. To our knowledge, no studies have reported the impacts of plant dietary fibre in domestic cats fed raw-red meat diets.

This study aimed firstly, to investigate the effects of plant dietary fibre inclusion to a complete and balanced raw red-meat diet on parameters such as faecal output, faecal score, faecal pH, faecal organic acid concentrations and the composition of the faecal bacteriome in the domestic cat. Secondly, we compared the bacteriome of cats fed the raw-diet supplemented with fibre to that from cats fed a commercially available kibble that contained a similar total dietary fibre. While the raw-diet supplemented with dietary fibre and the kibble diets had different macronutrient compositions, domestic cats are not fed individual dietary components, but rather a complete diet. Therefore, understanding how diet as a whole, affects the bacteriome, is an important step in unravelling the relationship between diet, microbiome, and health of the host.

We hypothesise that the addition of dietary fibre to a Raw diet will select for a bacteriome that more closely resembles that of cats fed a kibble diet, compared to cats the raw diet alone. We also hypothesised that these changes to the microbiome would consequently the effect organic acid profiles observed in faeces, with increased short chain fatty acid production from inclusion of dietary fibre in the Raw diet.

## Materials and methods

The protocol was approved by the Massey University Animal Ethics Committee (MUAEC 16/41) and all cats are housed at the Centre for Feline Nutrition (Massey University, Palmerston North, New Zealand). On conclusion of this study, the cats returned to their colony housing.

### Animals and diets

Twelve neutered, domestic short hair cats between 2–8 years of age were block randomised into three groups (four animals per group, balanced for gender and age) and fed according to a cross-over design. The test diets were; Raw beef (Raw), Raw beef with inulin (2% ‘as is’ inclusion; Orafti Synergy 1, Benuo, Belgium) and cellulose (2% ‘as is’ inclusion; Avicel, Hawkins Watts, New Zealand; Raw+Fibre), and a commercially available kibble (Optimum Adult, MARS Incorporated) (Table 1). All diets were formulated to meet Association of American Feed Control Officials [5] guidelines for adult maintenance, with a feline vitamin and mineral premix added to the Raw diets. Raw meat diets were stored at -20°C and defrosted in a fridge (3°C) for 24 hours before use. Once thawed, the raw meat was mixed and divided into two portions; one portion kept as raw meat, and dietary fibre added to the other at the levels stipulated above.

**Table 1:** Composition of test diets fed to domestic cats.

	Diet		
Component	*Kibble* ^*a*^	*Raw+Fibre* ^*b*^	*Raw* ^*c*^
Crude protein (% DM)	41.5	66.6	74.4
Crude fat (% DM)	16.1	15.4	19.0
Crude fibre (% DM)	1.8	3.51	0.9
Ash (% DM)	8.9	4.72	5.3
NFE^d^ (% DM)	31.8	9.78	0.4
Gross Energy (kj/g)	20.0	23.3	23.8
Total Dietary Fibre (% DM)	12.9	11.7	1.3
Soluble Dietary Fibre (% DM)	2.0	0.2	0.04
Insoluble Dietary Fibre (% DM)	11.0	11.5	1.2

Test diets (Kibble, Raw+Fibre and Raw) fed to adult domestic shorthair cats (n = 12) for 21-days in a crossover design, with a 21-day washout period between each test phase.

Ingredient List:

^a^ Poultry and poultry by-products, cereals, cereal protein, poultry digest, salt, beet pulp, minerals (potassium chloride, zinc sulphate, ferrous sulphate, copper sulphate, potassium iodide), vitamins (A, B1, B2, B3, B6, B9, B12, C, E and choline), methionine, taurine, antioxidants, inulin and yucca.

^b^73% beef muscle, 10% beef liver, 5% bone chip, 5% beef tripe, 3.5% beef heart, 3.5% beef kidney, 0.2% feline vitamin and mineral premix, 2% inulin (as is basis) and 2% cellulose (as is basis)–equating to 13.4% on a dry matter basis.

^c^ 73% beef muscle, 10% beef liver, 5% bone chip, 5% beef tripe, 3.5% beef heart, 3.5% beef kidney, 0.2% feline vitamin and mineral premix.

^d^ Nitrogen free extractives, calculated by difference (100 –crude protein–crude fat–crude fibre–ash)

Each diet was fed to maintenance energy requirements (100kcal/kg BW^67^) during each of the three 21-day experimental phases, where feeding was altered weekly according to MER at that body weight. There was a 21-day washout period between each feeding phase, when a commercial canned diet was fed *ad libitum*. During each experimental phase the cats were housed in individual cages (80cm x 80cm x 110cm). The cats were then returned to colony housing (1400 x 2400 x 1400cm) for the washout phase. Total intake and refusal were recorded daily for each cat during the experimental phase and a group average recorded during the washout phase. Water was available *ad libitum*. Body weight was measured weekly throughout the study. All cats were at their ideal body weight at the start of the trial, and this did not significantly alter at the end.

Total intake, total urine and total faecal output (stored at -20°C before analysis) were recorded twice daily (am and pm) over the final 5-day period (day 17–21) of each experimental phase. Faeces used for analysis were scored using a 5-point visual scale (1–5 scale whereby grade 1 is classified as ‘hard and dry’, and 5, watery diarrhoea [29]). A trained person was responsible for the scoring of all faecal samples. The pH of the last passed faeces in each five day period was measured by adding 20ml distilled water to 2g of faeces [30] using a pH probe (HandyLab 100, SI Analytics GmbH, Germany). Before analysis, the sample was homogenised, and one replicate used. A fresh faecal sample was also collected on day 15 of the test diet feeding phase within ten minutes of defecation for bacteriome and organic acid analysis. The sample was immediately frozen in liquid nitrogen and stored at -80°C until analysis.

### Laboratory analysis

#### Apparent macronutrient digestibility

Before analysis, diets were subsampled then homogenised. Faecal samples were freeze-dried, bulked according to individual cat (collection took place over 5 days) and ground. Both were analysed for moisture content using a convection oven at 105°C, and ash residue determined in a 550°C furnace (AOAC 930.15/925.10/942.05). Apparent macronutrient digestibility (fat, protein, ash and gross energy) of each diet was calculated as previously described [31]. Dry matter (DM) was calculated as 100, less the percentage moisture. Crude protein was determined using the Leco total combustion (Dumas) method (AOAC 968.06), and crude fat using acid hydrolysis/Mojonnier extraction (AOAC 954.02). Crude fibre was determined using the gravimetric method (AOAC 962.09/978.10) and gross energy (GE) was measured using bomb calorimetry. Nitrogen free extracts (NFE) were calculated by difference. Total dietary fibre, insoluble dietary fibre and soluble dietary fibre were determined using the Megazyme assay (AOAC 991.45). The above assays were performed in an analytical lab accredited to ISO 17025 through IANZ, New Zealand.

#### Faecal organic acids

Faecal samples were diluted 1:5 with phosphate-buffered saline (PBS) containing 2-ethylbutyric acid as an internal standard. Faecal aqueous extracts were analysed as described previously [32]. Briefly, aqueous extracts were acidified, phase separated into diethyl ether and stored at -80°C until analysis. Organic acids were derivatised with N-tert-butyldimethylsilyl-N-methyltrifluoroacetamide plus 1% tert-butyldimethylchlorosilane (MTBSTFA + TBDMSCI, 99:1; Sigma-Aldrich) and analysed on a Shimadzu capillary gas chromatography (GC) system (GC-2010 Plus, Tokyo, Japan) equipped with a flame ionization detector (FID) and fitted with a Restek column (SH-Rtx-1, 30 m × 0.25 mm ID × 0.25 μm) using helium as the carrier gas. The GC-FID was controlled and data processed using Shimadzu GC Work Station LabSolutions Version 5.3, with sample organic acids quantified in reference to authentic standards.

#### Faecal bacteriome

NucleoSpin Soil kits (Macherey-Nagel, Düren, Germany) were used to extract DNA from faecal samples according to the manufacturer’s instructions, with the addition of a bead beating step using a Mini-Beadbeater-96 (BioSpec Products, Bartlesville, OK, USA) set for four minutes. Faecal microbial profiles were determined by analysis of the V3 to V4 region of the bacterial 16S rRNA gene using Illumina MiSeq paired-end 2 x 250 bp amplicon sequencing [33]. The forward primer sequence was CCTACGGGNGGCWGCAG and the reverse primer sequence was GACTACHVGGGTATCTAATCC. Faecal microbial amplicon sequences were processed using QIIME 1.8 [34]. Reads were quality filtered using default settings and sequences were chimera-checked using the USEARCH method against the Greengenes database (release GG_13_8). Chimeric sequences were removed from subsequent analyses. Sequences were clustered at 97% similarity into operational taxonomic units (OTUs) using the UCLUST method. Representative sequences were assigned taxonomies using the RDP classifier, and OTU’s were then grouped according to taxonomic level (phylum, family, order, class and genus) for further analysis.

### Statistical analysis

Analysis of faecal organic acid profiles, faecal score, faecal output and faecal pH was completed using Linear Mixed Effects Model (REML) (GenStat version 18.1[35]. Carryover effect, ‘Phase’ and ‘Diet’ (Kibble, Raw+Fibre and Raw) were used as fixed effects and ‘Cat’ as a random effect. Faecal output data, lactate, acetate, gross energy, protein and fat apparent macronutrient digestibility were log transformed, to meet the assumptions of normality and homogeneity. Valerate, total SCFA [36] and total BCFA were square root transformed to also meet these assumptions. Molar ratios of faecal organic acids were analysed using ‘Phase’ ‘Diet’ as the fixed effects and ‘Cat’ as a random effect. Principle component analysis was performed to assess the variance of faecal organic acids. Body weight was analysed using repeated measures ANOVA. P < 0.05 was considered statistically significant, and P < 0.1 a trend.

The R mixOmics package was used to condense the dataset into families and genera which were numerically important using the “nearZeroVar” function which removed observed bacteria present in numbers below a set threshold (0.0001%). This provided the dataset for statistical analysis, and R statistical software (R version 3.3.3 [37]) was used for all bacterial analyses. Permutation ANOVA was used to determine differences between the relative abundance of taxa due to dietary treatment. Multivariate analysis integrating the faecal 16S rRNA bacterial data and faecal organic acid dataset, was performed using R mixOmics package [38]. Sparse Partial Least Squares (sPLS) regression was performed using canonical mode and correlations cut off was defined as > |0.6| to generate a network plot. Comparison of overall communities was performed using the ANOSIM function [39], an implementation of a non-parametric multivariate analysis of variance, from the vegan package for R.

## Results

### Apparent macronutrient digestibility

Body weight was not significantly different between phases (P = 0.463). Dry matter intake tended (P = 0.09) to be higher on the Kibble dietary treatment (66 g DM/d), compared to the Raw+Fibre (60.8 g DM/d) and Raw diets (60.3 g DM/d; pooled SEM 3.6). The apparent digestibilities of DM, GE, protein and fat were lower (P < 0.001) in the Kibble diet compared to th Raw and Raw+Fibre dietary treatments (Table 2).

**Table 2:** Apparent macronutrient digestibility of test diets.

	Diet				
Digestibility	*Kibble*	*Raw+Fibre*	*Raw*	Pooled SEM	P Value
Dry Matter %	79.56^c^	90.29^b^	93.79^a^	1.625	<0.001
Gross Energy %	80.49^b^	97.78^a^	98.44^a^	1.082	<0.001
Protein %	79.54^c^	96.74^b^	99.34^a^	1.087	<0.001
Fat %	91.01^c^	98.12^b^	99.64^a^	0.314	<0.001

Dry matter (DM), gross energy (GE), protein and fat digestibility of domestic cats fed Kibble, Raw+Fibre and Raw test diets to maintenance energy requirements, in a cross over design. Results are presented as mean and associated pooled standard error of the mean (SEM).

^ab^ Differing subscripts denote means with significant differences between diet groups (P < 0.05)

### Faecal score, output and pH

Faecal scores were higher in the Raw+Fibre and Kibble dietary treatments than the Raw (P = 0.002) (Table 3). Faecal output was greatest in the Kibble diet, both on an ‘as is’ and DM per day basis (P = 0.006 and P < 0.001 respectively; Table 3). Faecal pH was lower (P = 0.001) in Kibble compared to both Raw and Raw+Fibre dietary treatments (Table 3).

**Table 3:** Changes to faecal score, faecal output, and faecal pH when fed Kibble, Raw+Fibre and Raw diets.

	Diet				
	*Kibble*	*Raw+Fibre*	*Raw*	Pooled SEM	P Value
Faecal Score^1^	3.39^a^	3.46^a^	1.83^b^	0.290	0.002
Faecal Output (g/day)^2^	38.40^a^	23.69^b^	22.20^b^	4.529	0.006
Faecal Output (g/DM/day)	13.93^a^	8.08^b^	4.38^c^	7.176	<0.001
Faecal pH	6.18^b^	7.04^a^^b^	7.58^a^	0.218	0.001

Faecal score, output and pH of domestic cats fed Kibble, Raw+Fibre, and Raw diets. Results are presented with means and pooled standard error of the mean (SEM).

^1^ 1–5 scale whereby grade 1 is hard and dry faeces, and grade 5 is watery diarrhoea

^2^ Reported on an ‘as-is’ basis

^abc^ Differing subscripts denote means with significant differences between diet groups (P < 0.05)

### Faecal organic acids

**Fig 1:**
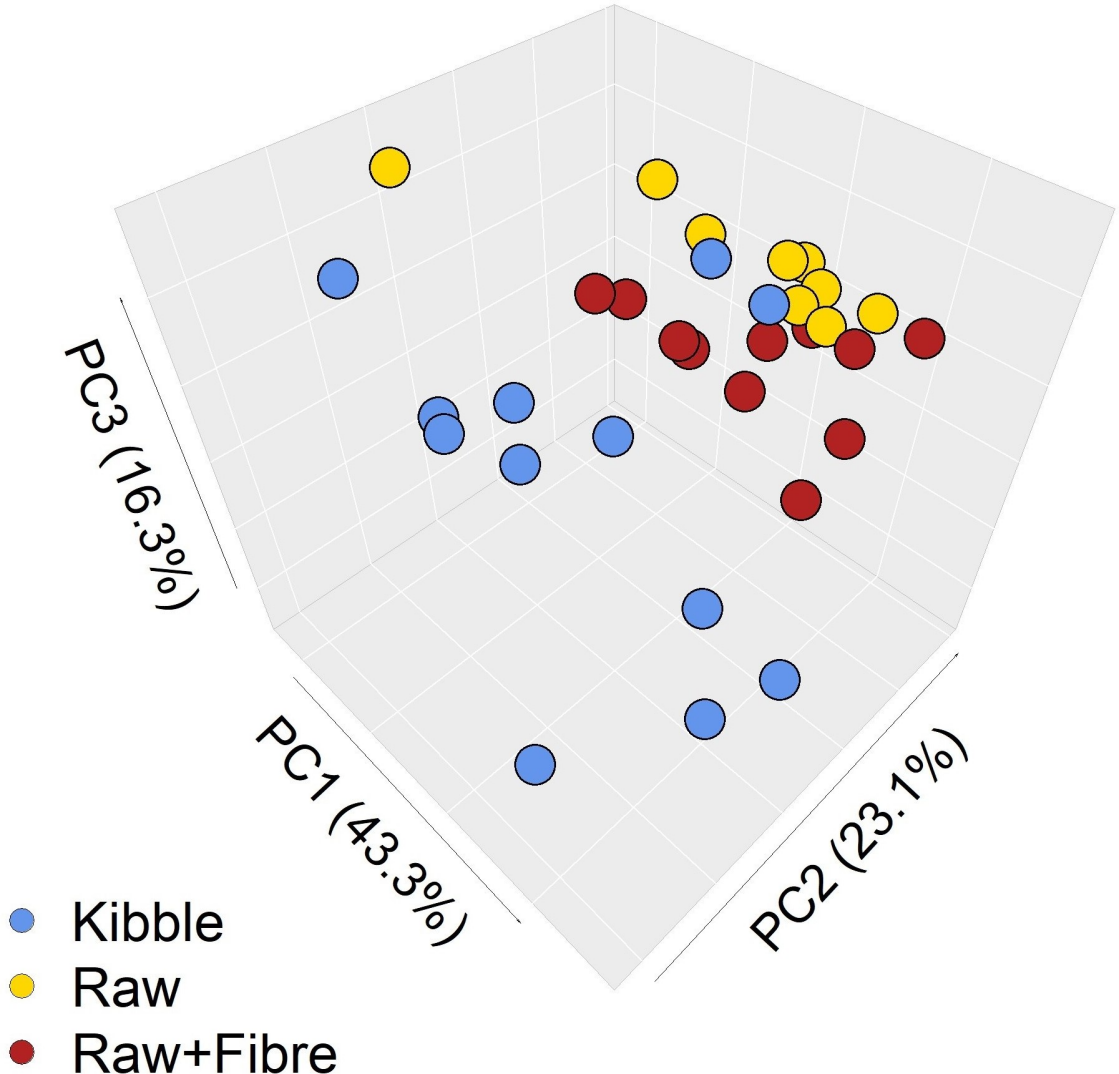
Principle-components analysis of the effect of diet on faecal organic acid profiles. PCA of faecal organic acid profiles from adult domestic cats fed Kibble (blue), Raw+Fibre (red) and Raw (gold) diets. Clustering according to dietary treatment is shown and highlights shifts in the overall organic acid profile.

Faecal concentration of propionate was lower on the Raw diet (P = 0.027), succinate higher on the Kibble (P < 0.001) and lactate higher on the Raw+Fibre dietary treatment (P = 0.031; Table 4). Faecal concentrations of acetate, butyrate, total SCFA and BCFA were not found to be significantly different (P > 0.05). Principle-component analysis showed organic acid profiles clustered according to diet (Fig 1).

**Table 4:** Faecal organic acid profiles of domestic cats fed Kibble, Raw+Fibre and Raw diets to maintenance energy requirements.

	Diet				
Organic acid (μmol/g DM faeces)	*Kibble*	*Raw+Fibre*	*Raw*	Pooled SEM	P Value
Acetate	196.37	141.74	123.84	37.122	0.392
Propionate	152.20^a^	105.60^a^	51.80^b^	22.950	0.027
Butyrate	67.13	53.80	49.40	15.210	0.736
Total SCFA^1^	528.08	364.05	296.18	78.592	0.157
Isobutyrate	11.10	10.97	13.28	3.545	0.915
Isovalerate	21.41	19.71	25.52	6.606	0.869
Total BCFA^2^	28.59	29.16	38.56	10.508	0.836
Valerate	59.08	21.58	49.25	22.703	0.405
Lactate	2.99^a^	6.32^a^	0.18^b^	2.928	0.031
Hexanoate^#^	4.96	1.88	2.06	2.064	0.378
Succinate^#^	15.46^a^	1.16^b^	0.48^b^	4.193	<0.001

Results are presented as mean and associated pooled standard error of the mean (SEM).

^#^ kruskal-wallis analysis completed due to lack of homogeneity of data

^1^ Total SCFA = acetate + propionate + butyrate + isobutyrate + isovalerate + valerate

^2^ Total BCFA = isobutyrate + isovalerate

^ab^ Differing subscripts denote means with significant differences between diet groups (P < 0.05)

As a proportion of total SCFA, the acetate:propionate:butyrate ratio was 47:35:18 (Kibble), 49:34:17 (Raw+Fibre) and 48:22:31 (Raw). The proportion of butyrate was highest in the Raw diet (P < 0.001), and propionate the lowest (P < 0.001).

### Faecal bacteriome

Resulting sequence reads were deposited in the NCBI Sequence Read Archive (SRA) and are publicly available under the accession number PRJNA432468.

#### Bacterial diversity

**Fig 2:**
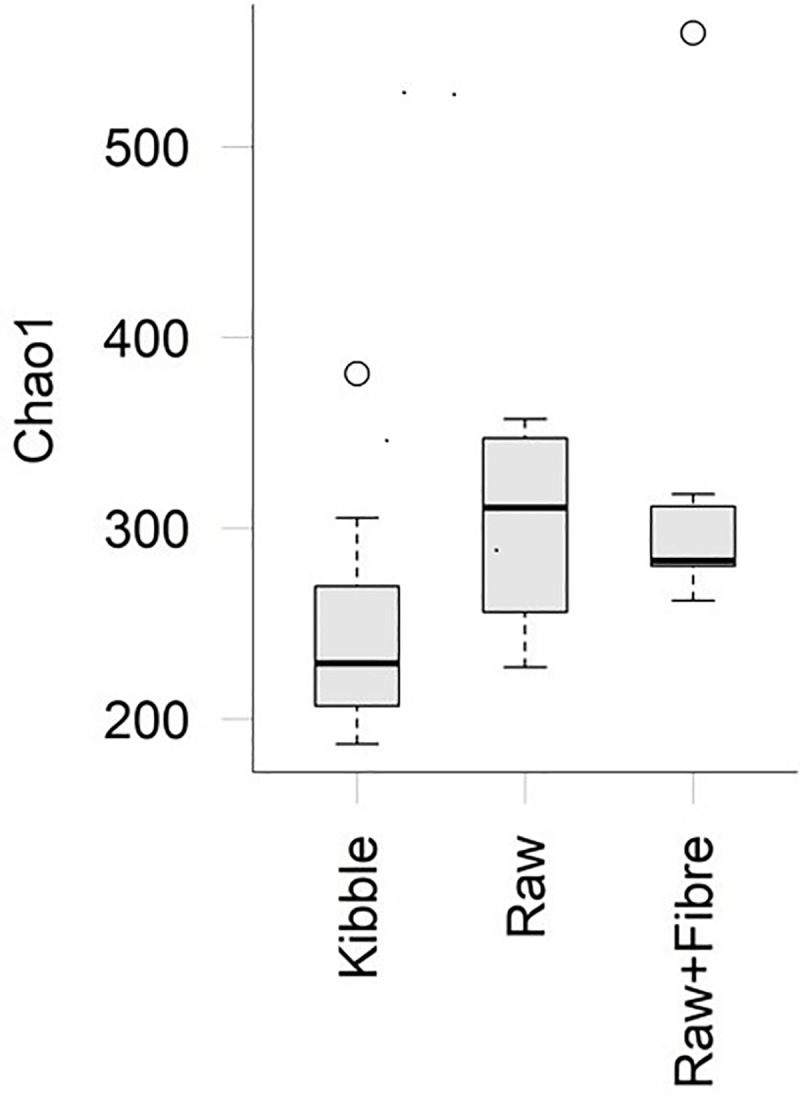
Chao 1 alpha diversity index boxplot. Alpha diversity of bacterial genera from adult domestic shorthair cats fed Kibble, Raw+Fibre and Raw diets. A slight trend (P = 0.08) for a decrease in Chao1 alpha diversity in the Kibble diet was observed. Circles denote outliers.

Assessment of alpha diversity (Chao 1 index) found that there was a trend (P = 0.08) for cats during the Kibble dietary treatment to have a lower faecal diversity than the Raw (Fig 2). The Raw+Fibre dietary treatment resulted in an intermediate level of alpha diversity to the Raw and Kibble.

#### Bacteriome composition

A total of 51 bacterial taxa at the genera or higher level were identified in the current study (S1 Table). The five most abundant taxa in the Kibble dietary treatment were *Prevotella* (39.7% of sequence reads), *Unclassified Peptostreptococcaceae* (18.5% of sequence reads), *Megasphaera* (10.3% of sequence reads), *Blautia* (4.7% of sequence reads) and *Unclassified Lachnospiraceae* (4.4% of sequence reads). The most abundant taxa in the Raw+Fibre dietary treatment were *Unclassified Peptostreptococcaceae* (25.5% of sequence reads), *Prevotella* (13.6% of sequence reads), *Clostridium* (8.8% of sequence reads), *Blautia* (7.8% of sequence reads) and *Unclassified Lachnospiraceae* (7% of sequence reads). *Clostridium* (24.7% of sequence reads) was the most abundant on the Raw dietary treatment, followed by *Unclassified Peptostreptococcaceae* (18.5% of sequence reads), *Fusobacterium* (12.6% of sequence reads), *Unclassified Prevotellaceae* (7.5% of sequence reads) and *Unclassified Clostridiales* (5.7% of sequence reads).

**Fig 3:**
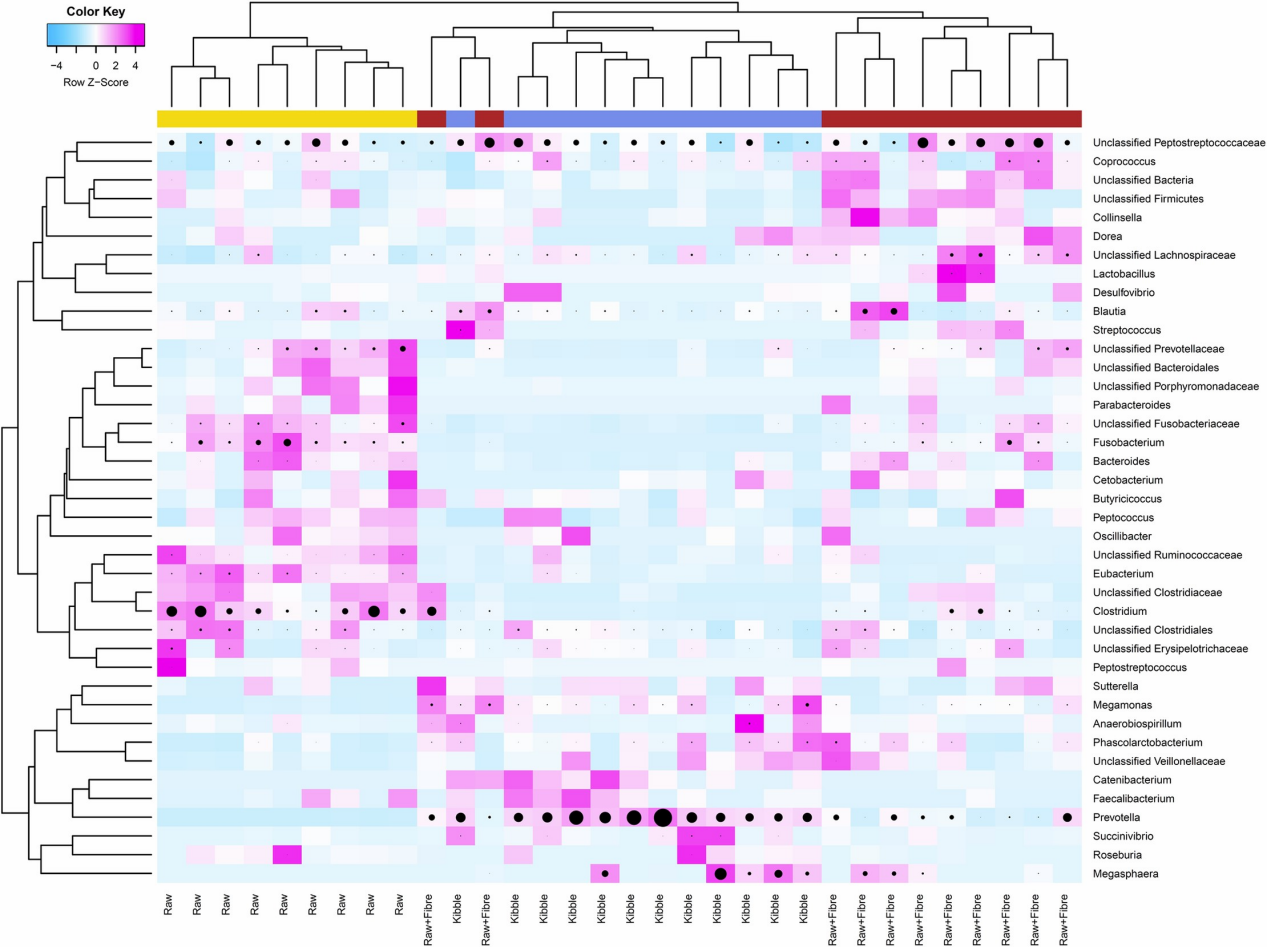
Heat map showing hierarchical clustering of bacterial relative abundances. Bacterial taxa are shown at the genus level from in the faecal bacteriome of adult domestic shorthair cats fed Kibble, Raw+Fibre and Raw diets. Heat map colours indicate normalized (Z score) relative abundance of each genus scaled across rows. Intensity of magenta colour denotes number of standard deviation above the mean and intensity of blue colour denotes number of standard deviation below the mean. Black circles show relative abundance of each taxa without scale normalization, with size of circle proportional to relative abundance. Colour ribbon at the top of the figure indicates diet; Raw (gold), Kibble (blue), and Raw+Fibre (red).

Comparison of communities using permutation ANOVA found that 31 taxa had significantly different relative abundances between dietary treatments (Table 5; Fig 3). The Kibble dietary treatment had significantly higher proportions of *Asaccharobacter*, *Prevotella*, *Catenibacterium* and *Succinivibrio* (Permutation ANOVA FDR < 0.05). The Raw diet had highest proportions of *Clostridium*, *Eubacterium* and *Fusobacterium* and the Raw+Fibre dietary treatment, the cats had highest proportions of *Bifidobacterium*, *Colinsella* and *Lactobacillus*.

**Table 5:** Bacterial taxa (proportion of total sequences) in the faecal bacteriome of domestic cats fed Kibble (n = 12), Raw+Fibre (n = 11) and Raw (n = 9) diets. Only significant interactions (P < 0.05) analysed by permutation ANOVA are reported. Fishers-Protected Least Significant Difference analysis was then used directly comparing dietary treatment. False Discovery Rate (FDR) indicates multiple testing adjusted P value.

Phyla	Family	Genus	Diet							
			Kibble		Raw+Fibre		Raw			
			Mean	SEM	Mean	SEM	Mean	SEM	P value	FDR
Actinobacteria	Bifidobacteriaceae	*Bifidobacterium*	0.000^b^	0.0000	0.116^a^	0.0630	0.000^b^	0.0000	0.0182	0.0420
	Coriobacteriaceae	*Asaccharobacter*	0.047^a^	0.0135	0.003^b^	0.0033	0.002^b^	0.0018	0.0002	0.0012
		*Collinsella*	0.026^b^	0.0099	0.139^a^	0.0421	0.032^b^	0.0106	0.0000	0.0000
Bacteroidetes	Bacteroidaceae	*Bacteroides*	0.222^b^	0.0854	1.040^a^	0.2913	1.571^a^	0.4182	0.0068	0.0194
	Other	*Uncl*. *Bacteroidales*	0.013^b^	0.0070	0.066^b^	0.0221	0.181^a^	0.0592	0.0056	0.0174
	Porphyromonadaceae	*Uncl*. *Porphyromonadaceae*	0.002^b^	0.0019	0.027^b^	0.0139	0.183^a^	0.0823	0.0026	0.0120
		*Parabacteroides*	0.000^b^	0.0000	0.053^a^^b^	0.0331	0.150^a^	0.0625	0.0058	0.0174
	Prevotellaceae	*Uncl*. *Prevotellaceae*	0.921^b^	0.4902	4.116^a^^b^	1.1752	7.476^a^	2.1490	0.0040	0.0150
		*Prevotella*	39.710^a^	3.0888	13.559^b^	3.0276	0.110^c^	0.0597	0.0000	0.0000
	Other	*Uncl*. *Bacteroidetes*	0.003^b^	0.0027	0.010^a^^b^	0.0072	0.026^a^	0.0087	0.0446	0.0863
Firmicutes	Lactobacillaceae	*Lactobacillus*	0.000^b^	0.0000	0.960^a^	0.5050	0.016^b^	0.0164	0.0028	0.0120
		*Uncl*. *Lactobacillaceae*	0.000^b^	0.0000	0.038^a^	0.0227	0.000^b^	0.0000	0.0364	0.0728
	Clostridiaceae	*Clostridium*	0.346^c^	0.2041	8.815^b^	2.9814	24.694^a^	4.1243	0.0000	0.0000
		*Uncl*. *Clostridiaceae*	0.015^c^	0.0111	0.254^b^	0.0823	0.542^a^	0.0933	0.0000	0.0000
	Eubacteriaceae	*Eubacterium*	0.554^b^	0.2243	0.405^b^	0.2159	4.394^a^	0.6663	0.0000	0.0000
	Lachnospiraceae	*Uncl*. *Lachnospiraceae*	4.419^b^	0.5222	7.048^a^	1.1774	3.090^b^	0.7969	0.0090	0.0245
	Peptostreptococcaceae	*Peptostreptococcus*	0.003	0.0028	0.042	0.0307	0.218	0.1567	0.0363	0.0728
	Ruminococcaceae	*Faecalibacterium*	0.082^a^	0.0237	0.003^a^	0.0034	0.044^a^^b^	0.0228	0.0172	0.0413
		*Uncl*. *Ruminococcaceae*	0.498^b^	0.1897	0.359^b^	0.1695	2.224^a^	0.4948	0.0000	0.0000
		*Subdoligranulum*	0.102	0.0569	0.000	0.0000	0.000	0.0000	0.0316	0.0702
	Veillonellaceae	*Allisonella*	0.056^a^	0.0179	0.025^a^^b^	0.0148	0.000^b^	0.0000	0.0345	0.0728
		*Megamonas*	3.998^a^	0.9283	3.287^a^	0.9476	0.180^b^	0.0931	0.0054	0.0174
		*Uncl*. *Veillonellaceae*	0.291^a^	0.0631	0.231^a^	0.0765	0.038^b^	0.0177	0.0154	0.0385
		*Phascolarctobacterium*	2.845^a^	0.5585	2.369^a^	0.6374	0.601^b^	0.2704	0.0128	0.0334
	Other	*Uncl*. *Clostridia*	0.000^b^	0.0000	0.000^b^	0.0000	0.047^a^	0.0189	0.0000	0.0000
	Erysipelotrichaceae	*Catenibacterium*	0.163^a^	0.0467	0.035^b^	0.0231	0.000^b^	0.0000	0.0008	0.0040
	Other	*Uncl*. *Firmicutes*	0.021^b^	0.0084	0.140^a^	0.0343	0.083^a^^b^	0.0265	0.0046	0.0162
Fusobacteria	Fusobacteriaceae	*Fusobacterium*	0.028^c^	0.0131	4.848^b^	1.4057	12.584^a^	2.2270	0.0000	0.0000
		*Uncl*.*Fusobacteriaceae*	0.465^c^	0.1409	2.310^b^	0.5485	5.039^a^	1.0273	0.0000	0.0000
	Other	*Uncl*. *Bacteria*	0.372^b^	0.0364	0.826^a^	0.1246	0.486^b^	0.0843	0.0008	0.0040
Proteobacteria	Succinivibrionaceae	*Succinivibrio*	1.183^a^	0.4204	0.144^b^	0.0596	0.067^b^	0.0481	0.0036	0.0144

Uncl = unclassified

^abc^ Differing subscripts denote significant differences between means if dietary treatments

### Data integration

**Fig 4:**
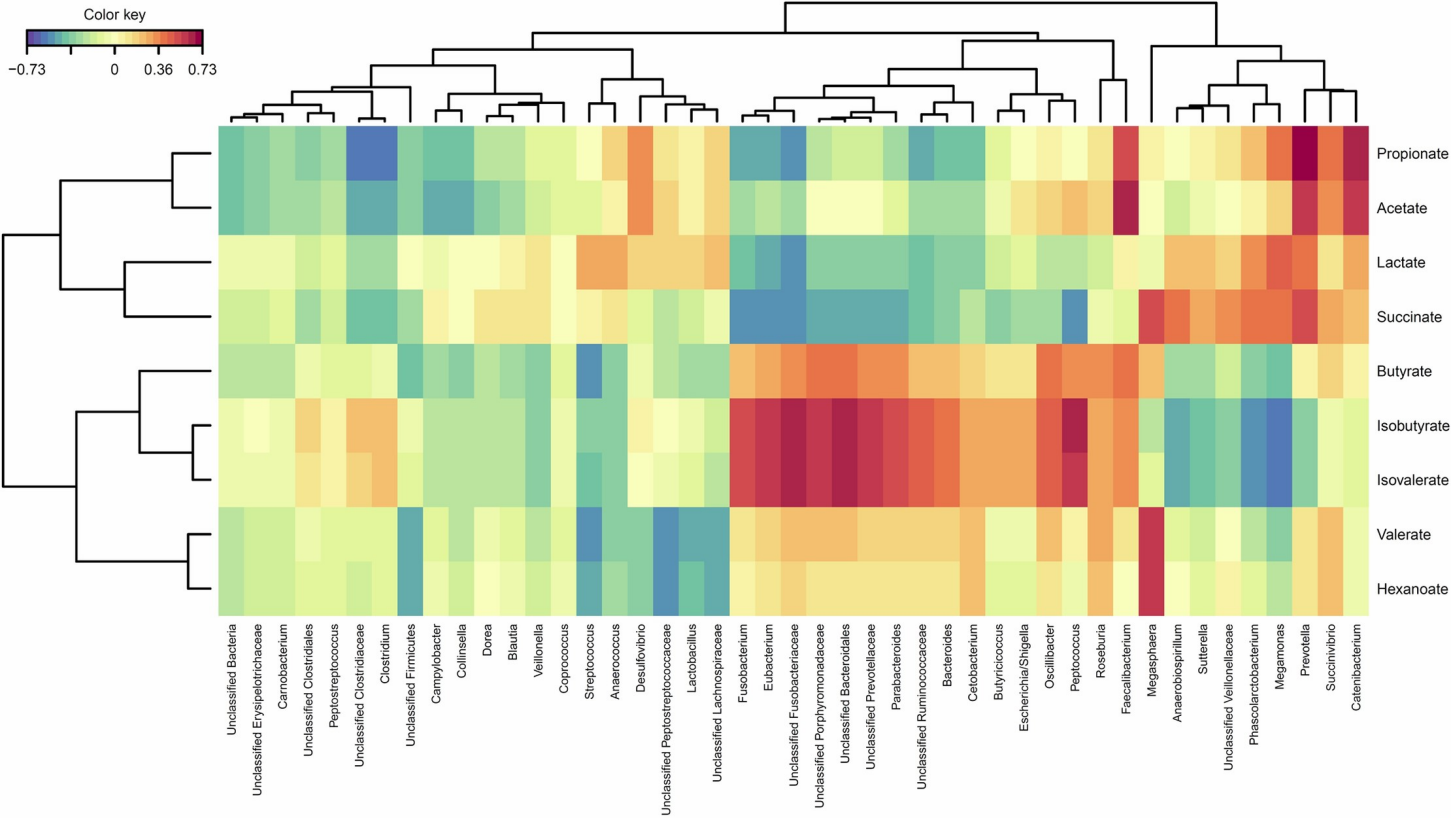
Canonical correlation clustered image map illustrating associations between organic acid concentrations and bacterial genus. Faecal organic acid concentrations (umol/g DM faeces) from faeces of adult domestic cats fed three experimental diets (Kibble, Raw+Fibre and Raw). Correlation cut off was |0.6|, greater that 0.6 considered a highly positive correlation (increasing red intensity) and lower than -0.6 considered a highly negative correlation (increasing blue intensity).

**Fig 5:**
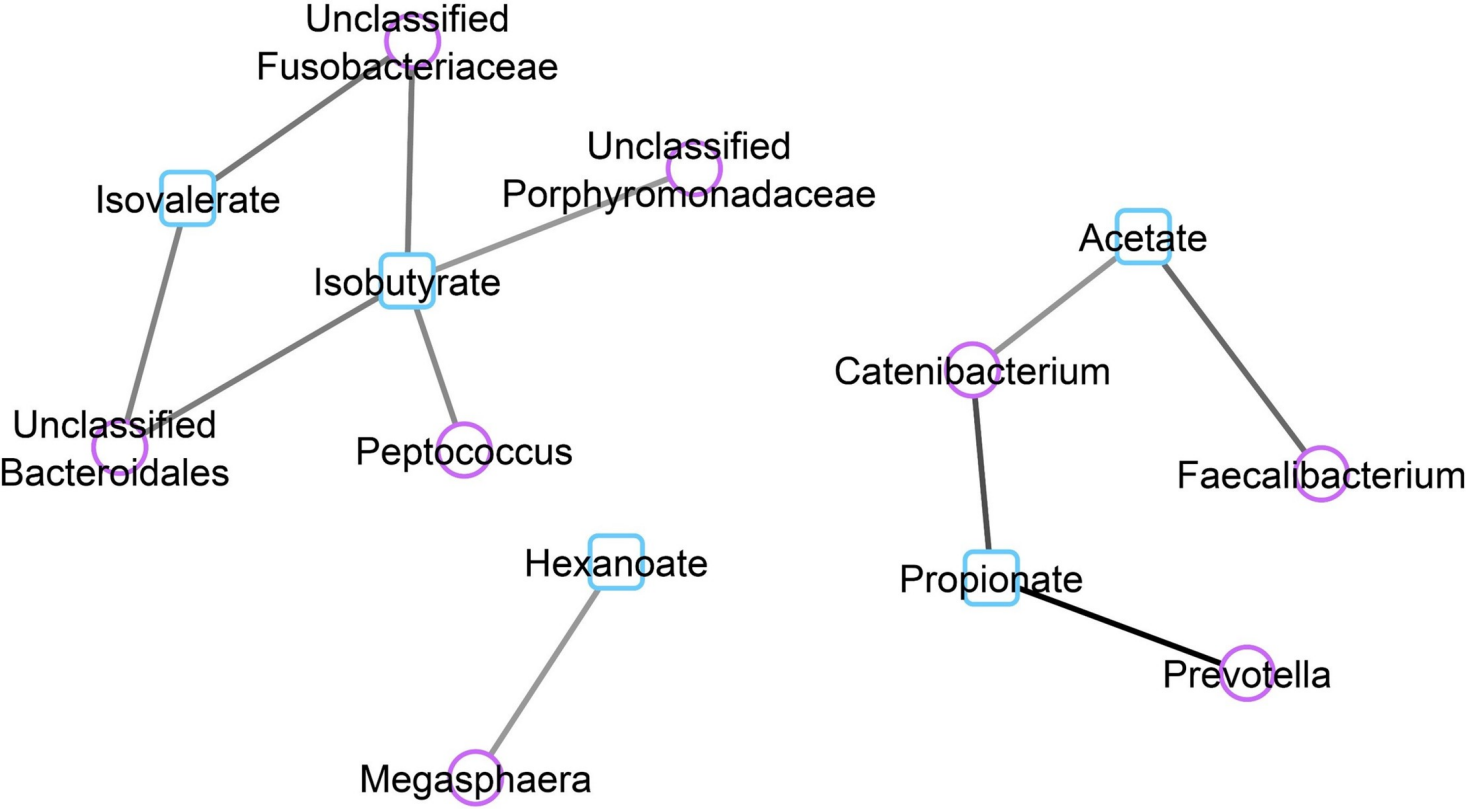
Canonical correlation network plot illustrating relationships between bacterial taxa and organic acid concentrations. Samples from faeces of cats fed Kibble, Raw and Raw+Fibre diets. Relationships cut off at >|0.6|. Purple circles denote bacterial taxa and blue squares denote organic acids. Intensity of grey/black line denotes strength of positive correlation.

A canonical correlation Clustered Image Map (CIM) illustrates relationships between faecal organic acid profiles and the bacterial genera observed (Fig 4). A corresponding network plot (using canonical correlation cut off of 0.6; Fig 5) identifies highly positive correlations were observed between acetate concentrations and the presence of *Faecalibacterium* and *Catenibacterium* while propionate was correlated with *Prevotella* and *Cantenibacterium*. Isobutyrate was highly correlated with *Peptococcus*, *Unclassified Porphyromonadaceae*, *Unclassifed Bacteroidales and Unclassifed Fusobacteriaceae* while the latter two families were highly correlated with isovalerate. Hexanoate concentrations was positively correlated with *Megasphaera*.

## Discussion

This study shows that the addition of plant dietary fibre to a complete and balanced raw red meat diet alters faecal pH, faecal output, faecal score, faecal bacteriome composition, and faecal organic acid profiles in the domestic cat. This supports our hypothesis, that faecal characteristics become more similar to those from Kibble compared to Raw dietary treatment when dietary fibre is added.

### Apparent macronutrient digestibility

The Raw diet had higher apparent digestibility of DM, GE, protein and fat in this study, in agreement with other studies conducted in domestic cats [40, 41], sand cats [42], and domestic dogs [20]. Previous studies have shown that fibre reduces digestibility *in vivo* [43], and *in vitro* [44]. This is most likely due to physical inhibition or the properties of the dietary fibre, such as gel-forming and water binding capacities. However, it is interesting to note the inclusion of 10% dietary fibre (DM basis) did not decrease DM digestibility by a similar amount. Instead, DM digestibility was only decreased by 3%, which suggests that even an obligate carnivore like the domestic cat can harbour a gut bacteriome that efficiently utilises non-digestible carbohydrates.

### Faecal quality

Faecal quality is an important factor for pet owners, and was assessed in this study by evaluating faecal score and output. The faecal scoring system used in this study considers a grade 1.5–2.5 on a 5 point scale is ideal [29]. We observed that faecal score was improved in the Raw dietary treatment (1.8/5 *vs*. 3.4/5 in the Raw *vs*. Raw+Fibre- diets, respectively). The cats fed fibre-containing diets (Kibble and Raw+Fibre) defecated 3-times more frequently and produced a larger volume of faeces, despite a relatively similar DM intake (averaging 60 g DM/day). The decrease in faecal output and lower number of defecations observed with the Raw diet is most likely due to the highly digestible nature of the diet. In captive exotic felids, Kerr et al [16] found that the addition of cellulose to raw beef increased faecal output and decreased faecal scores when compared to beet pulp, similar to what we observed in this study with domestic cats. Inulin is known to increase defecation frequency in humans [45] and cellulose is known to decrease intestinal transit time [46] and it appears that, based on these results, they have similar impacts in the domestic cat.

### Faecal bacteriome and fermentation end products

*Prevotella*, *Unclassified Peptostreptococcaceae* and *Megasphaera*, known fermenters of complex carbohydrates [47, 48], were the dominant genera observed in Kibble diet, comprising 68% of the taxa observed. In contrast, the relative abundance of *Clostridium*, *Unclassified Peptostreptococcaceae* and *Fusobacterium*, dominated the faeces in the Raw dietary treatment (*c*.55% of the total taxa observed). The addition of dietary fibre to the Raw diet indicated a profile intermediate of the two, with *Unclassified Peptostreptococcaceae*, *Prevotella* and *Clostridium* comprising almost 50% of the observed taxa. These observations are largely in agreement with previous studies investigating the impacts of dietary levels of carbohydrate/protein in the cat [17, 49] and dogs fed raw red meat diets [15, 20]. Although the bacteriome profiles in the Raw+Fibre dietary treatment were an intermediate between the Raw and Kibble diets, the faecal organic acid profiles from Raw+Fibre diet were more similar to those from the Kibble diet.

Thirty one bacterial taxa were affected by diet. The relative abundances of *Clostridium* and *Fusobacterium* were increased on the Raw diets, with the addition of dietary fibre reducing their relative abundance. Both *Fusobacterium* and *Clostridium* are a large, functionally diverse taxa, which typically degrade protein [50], and are associated with high protein diets in both the dog [15, 20] and cat [51]. The abundance of *Prevotella* increased greatly in the fibre-containing diets (*c*. 26% of sequence reads) compared to the Raw diet (0.11% of sequence reads). Comparison between the Kibble and Raw+Fibre faecal microbiome must consider the shift in crude protein and NFE content. There was a 25% (DM basis) decrease of crude protein and 21% increase in NFE on the Kibble diet. The changes to these macronutrient profiles are reflected in the microbiome, whereby *Prevotella* (prominent carbohydrate utilisers) increased from 13% on the Raw+Fibre diet, to 39% in the Kibble, suggesting that the increased dietary fibre content is driving this change. As taxa such as these increase, the change in resources (and decreased protein reaching the large intestine for bacterial fermentation) will subsequently reduce amino acid utilisers such as *Clostridium* and *Fusobacterium*, which we see in our data.The relative abundance of *Bifidobacterium* and *Lactobacillus* was higher in the Raw+Fibre diet compared to both the Kibble and Raw diets (0.1% and 0.9% respectively, in comparison to 0% in the Raw and Kibble diets). It has been shown that high protein diets decrease the relative abundance of *Bifidobacterium* [52]. Inulin is known to increase the abundance of *Bifidobacterium* in humans [53, 54], although this has not previously been linked specifically, to the inclusion of inulin in diets for cats or dogs. Kanakupt et al [55], however, did observe increased *Bifidobacterium* during supplementation of extruded diets with short chain fructooligosaccharide, an inulin derivative. Increased abundance of *Lactobacillus* associated with inulin has been observed in dogs [15]. Both bacterial taxa are known to be present in the faeces of healthy cats fed commercially available diets [56]. Other fermentable plant dietary fibre sources, such as yeast cell wall extract and beet pulp, have been shown to increase the relative abundance of these genera in dogs [15, 57], however cellulose alone has not. Both inulin and cellulose were included in the Raw+Fibre diet, although it appears that inulin (or a fermentable fibre source) provided the best substrate for these bacteria. These changes were not observed in the Kibble diet in this study, despite also containing inulin (albeit in a smaller amount). Both *Bifidobacterium* and *Lactobacillus* have been extensively studied in the human literature as they are known to increase in abundance in response to prebiotics, and specifically inulin [53, 58]. Therefore, their increased abundance in the Raw+Fibre diet may suggest beneficial effects of including a fermentable fibre source in raw meat diets for the domestic cat.

Analysis of faecal organic acid profiles found that there were few differences associated with diet, despite the large differences in dietary macronutrient profiles and shifts in the microbiome. However, faecal organic acids clustered according to diet, indicating that despite the lack of statistical differences for individual acids, changes occurred to the organic acid profile as a whole. Interestingly, the faecal acetate:propionate:butyrate ratio of the Raw+Fibre dietary treatment was almost identical to that of the Kibble. This similarity suggests that despite differences in the bacteriome composition, the fermentation processes or pathways may be similar between faecal bacteriomes from Raw+Fibre and Kibble dietary treatments. To gain a better insight into potential relationships between particular taxa and organic acids, we explored patterns of correlations between the two data sets. Genera that were highly positively correlated with organic acid production included *Prevotella*, *Catenibacterium*, *Faecalibacterium* and *Megasphaera*. With the exception of *Prevotella*, the abundances of these other genera were low (0.23–4.3% total sequence reads in Raw and Kibble diets, respectively). This observation raises the possibility that taxa with low relative abundance, may have the ability to cause a disproportionate change in the colonic environment. Understanding the absolute concentrations of organic acids present in the faecal matter may provide further insight. *Megasphaera* is known to utilise two substrates, glucose and lactate, depending on their availability in the colon [59]. In this study, it was found to be highly correlated with hexanoate production which suggests utilisation of glucose, and may explain the increased hexanoate production and its relative abundance on the Kibble diet.

In the current study, *Prevotella* and *Catenibacterium* were highly correlated with propionate production. Propionate was found to significantly alter according to dietary treatment, with Raw+Fibre and Kibble having similar faecal concentrations, and lower concentrations in the Raw diet. *Prevotella* and *Catenibacterium* decreased during the Raw dietary treatment, and may partially explain the low levels of propionate. *Prevotella* are commonly associated with increased amounts of dietary fibre consumed by humans [47]. Certain species of *Prevotella* have also been shown to produce succinate in mice models [60] which could then be metabolised to propionate; this may explain the correlation observed in the current study. There are three pathways that produce propionate; the succinate pathway from hexose sugars, propanediol pathway from deoxy sugars (such as fructose) and acrylate pathway via utilisation of lactate [61]. *Catenibacterium* are able to ferment carbohydrates and are part of the *Clostridium* subphylum cluster XVII [62]. Typical fermentative end products are acetate, butyrate, lactate and isobutyrate, when isolated from human faeces [63]. *Catenibacterium* cannot directly produce propionate, however they can produce lactate which can be converted to propionate via the acrylate pathways [61], thereby explaining the correlations observed in the current study.

Diet did not affect the concentration nor proportion of acetate in faeces in the current study. However, *Faecalibacterium* and *Catenibacterium* showed strong correlations with acetate concentrations in the faeces. While a proportion of acetate is absorbed, it can also be utilised by intestinal bacteria as an energy source, and the amount present in the faeces does not provide information as to its production or utilisation in the colon. For example, *Faecalibacterium prausnitzii* are able use acetate to produce butyrate [64].

Despite large differences in dietary macronutrient profiles, the concentration of butyrate was unaffected by diet; however when examined as a proportion of total SCFA, the molar ratio of butyrate was found to be greater in the Raw than the Kibble and Raw+Fibre dietary treatments. Butyrate production occurs through four main pathways and is controlled by two main enzymes. Butyryl-CoA:acetate CoA transferase (but) controls the acetyl CoA and gluterate pathways whereas butyrate kinase (buk) controls the 4-amino butyrate and lysine pathways [65]. A wide variety of bacteria have been shown to produce butyrate, many of which are of the phyla Firmicutes, such *Clostridium*, *Fusobacterium* and *Eubacterium* [66], which were abundant in the faeces of the cats fed Raw diet. Typically, butyrate is produced from carbohydrate fermentation [67] however, it can be synthesised from protein sources including specific amino acids and mucins [68]. Indeed, Fig 4 showed butyrate clustering with the typical products of amino acid fermentation (isobutyrate and isovalerate), instead of the carbohydrate fermenters, suggesting that in this study, butyrate may have been produced from amino acid fermentation (such as *Oscillibacter* and *Faecalibacterium*).

Previous studies have identified lactate-utilising and butyrate-producing bacteria in human faeces, all of whom were from the Clostridal cluster XIVa [69]. This can be seen in Fig 4, where lactate is generally negatively associated with the bacteria which are positively correlated with butyrate production, such as *Eubacterium* and *Fusobacterium*. This suggests that butyrate production may be abundant in the Raw diet, due the available substrate and conversion from lactate. Whilst predominantly being converted to butyrate, lactate has also been shown to be readily converted to propionate and valerate in humans [70], consistent with the low concentrations of lactate present in the faeces of the Raw diet in the current study. The higher concentration of lactate in the Kibble and Raw+Fibre dietary treatments is most likely due to the greater amount of rapidly fermentable material present in the colon [69].

There were no significant differences in individual, or total, BCFA faecal concentrations. This is of interest, as the increased protein content of the Raw diet, compared to the Kibble, was predicted to have greater concentrations of protein fermentation end products. This may be due to the amount of protein reaching the colon, as the Raw diet was so highly digestible, and the Kibble diet far lower (99% and 79% respectively). Isobutyrate was positively correlated with relative abundance of *Peptococcus* which is a known amino acid fermenter [71] and had the highest relative abundance in the Raw diet (0.3% of observed taxa). Isobutyrate was also highly correlated with *Unclassified Porphyromonadaceae*, *Unclassifed Bacteroidales* and *Unclassifed Fusobacteriaceae*, and isovalerate with the latter two taxa. Although the physiological implications for altered branched chain fatty acids (BCFAs), such as isobutyrate and isovalerate, remain largely unknown, there is some evidence that BCFAs can decrease the rate of *de novo* lipidogenesis in adipocytes, *in vitro* [28]. Therefore, it could be hypothesised that higher BCFAs could be metabolically beneficial when there is over-consumption of fat. However, whether this would occur *in vivo*, given the low overall concentrations of faecal BCFAs, remains to be determined.

## Conclusion

This study provides an insight into the effects of feeding raw meats diets with and without added dietary fibre to a domestic obligate carnivore, the cat. The results show that dietary fibre inclusion into a raw meat diet altered the faecal parameters assessed, bringing them closer to those produced by feeding a kibble diet. Associations between faecal bacteriomes and organic acid profiles from the different diets suggests complex cross-feeding may occur within the gastrointestinal microbiome. Alpha diversity of the Raw diet was not significantly lower than that of the Kibble and despite shifts in the microbiome, significant changes to individual organic acid concentrations were not observed. Although the health consequences of changes in organic acids, microbial community composition and dietary fibre requirement remains to be determined in cats, our data provides a foundation for further, more in-depth, research, assessing raw meat feeding regimes and their effect on domestic cat health.

## Supporting information

S1 Table Bacterial taxa observed in faeces of domestic cats fed Kibble, Raw+Fibre and Raw diets according to a 21-day block cross-over design. (XLSX)
